# Real-time evaluation and adaptation to facilitate rapid recruitment in a large, prospective cohort study

**DOI:** 10.1186/s12913-024-10750-5

**Published:** 2024-03-13

**Authors:** Ashley Honushefsky, Eric S. Wagner, Kathleen Sheridan, Kathleen M. Spickard, William R. LeMasters, Carroll N. Walter, Taryn Beaver, Anne Marie Lennon, Nickolas Papadopoulos, Alanna Kulchak Rahm, Adam H. Buchanan

**Affiliations:** 1 Geisinger, 549 Fair Street, Bloomsburg, PA 17815 USA; 2 The Johns Hopkins Institutions, Baltimore, MD USA

**Keywords:** Adaptations, Implementation outcomes, Rapid recruitment, Large cohort, Protocol adherence, Positive participant experience, Group consenting

## Abstract

**Background:**

Recruiting large cohorts efficiently can speed the translation of findings into care across a range of scientific disciplines and medical specialties. Recruitment can be hampered by factors such as financial barriers, logistical concerns, and lack of resources for patients and clinicians. These and other challenges can lead to underrepresentation in groups such as rural residents and racial and ethnic minorities. Here we discuss the implementation of various recruitment strategies for enrolling participants into a large, prospective cohort study, assessing the need for adaptations and making them in real-time, while maintaining high adherence to the protocol and high participant satisfaction.

**Methods:**

While conducting a large, prospective trial of a multi-cancer early detection blood test at Geisinger, an integrated health system in central Pennsylvania, we monitored recruitment progress, adherence to the protocol, and participants’ satisfaction. Tracking mechanisms such as paper records, electronic health records, research databases, dashboards, and electronic files were utilized to measure each outcome. We then reviewed study procedures and timelines to list the implementation strategies that were used to address barriers to recruitment, protocol adherence and participant satisfaction.

**Results:**

Adaptations to methods that contributed to achieving the enrollment goal included offering multiple recruitment options, adopting group consenting, improving visit convenience, increasing the use of electronic capture and the tracking of data and source documents, staffing optimization via leveraging resources external to the study team when appropriate, and integrating the disclosure of study results into routine clinical care without adding unfunded work for clinicians. We maintained high protocol adherence and positive participant experience as exhibited by a very low rate of protocol deviations and participant complaints.

**Conclusion:**

Recruiting rapidly for large studies – and thereby facilitating clinical translation – requires a nimble, creative approach that marshals available resources and changes course according to data. Planning a rigorous assessment of a study’s implementation outcomes prior to study recruitment can further ground study adaptations and facilitate translation into practice. This can be accomplished by proactively and continuously assessing and revising implementation strategies.

**Supplementary Information:**

The online version contains supplementary material available at 10.1186/s12913-024-10750-5.

## Background

Recruiting large cohorts in a relatively short period of time is essential to speed the translation of findings into care across a range of scientific disciplines and medical specialties. Rapid enrollment of large cohorts is important in precision medicine studies that require substantial cohorts to achieve the power necessary to compare clinical outcomes, including multi-cancer early detection (MCED) studies with regulatory implications and studies that seek to elucidate associations between genes and disease [1–5]. For example, the *All of Us* Research Program is seeking to enroll at least one million diverse participants [6]. Additionally, the National Health Service-Galleri Trial of an MCED test successfully enrolled 140,000 participants in 10 months [4]. These achievements highlight two key obstacles that researchers commonly face: recruiting underserved and underrepresented socioeconomic groups into large research studies, and recruiting on a large scale without the coordination of a national health service.

In addition to these challenges, factors such as language, financial barriers, logistical concerns, and a lack of resources for patients can prevent efficient enrollment [7]. For instance, studies enrolling older adults must overcome common recruitment challenges such as patients being too sick (too many comorbidities), patients’ families advising against participation, lack of interest, and transportation issues [8]. Recruiting among rural populations also presents distinct challenges such as geographic isolation, unique cultural and social aspects of rural settings, low population density, limited transportation, and limited access to technology, including high-speed internet [9]. Establishing procedures prior to trial start-up can maximize the success of trial implementation [10]. However, challenges arising throughout implementation may still threaten recruitment, retention, adherence to study design, and data collection, thus negatively impacting fulfillment of study aims. Therefore, the ability to identify and address such challenges in real-time is critical.

The DETECT-A study is the first interventional study of a blood-based MCED test. This test, called CancerSEEK, was used to detect cancers in older women without a history of cancer [11]. CancerSEEK screened for eight different cancer types (ovarian, liver, stomach, pancreas, esophagus, colorectum, lung, and breast cancers), which account for more than 60% of cancer deaths [11]. Participants had a blood draw, and those with a positive MCED test proceeded to whole body PET-CT scan for confirmation and localization of the tumor [11]. Enrollment and recruitment for DETECT-A was conducted at Geisinger, a single, partially integrated healthcare system spanning 45 counties in Pennsylvania, 35 of which are designated as rural. Geisinger was chosen as the recruitment site due to several features that were anticipated to facilitate recruitment.

By virtue of its large, stable, aging patient population and 25-year use of an electronic health record that can be queried for potentially eligible individuals [5, 12], investigators hoped to enroll 10,000 participants in 18 months. The purpose of this manuscript is to discuss the implementation of various recruitment strategies for enrolling participants into a large, prospective cohort study, assessing the need for adaptations and making them in real-time, while maintaining high adherence to the protocol and high participant satisfaction.

## Methods

### DETECT-A study overview

**Fig. 1:**
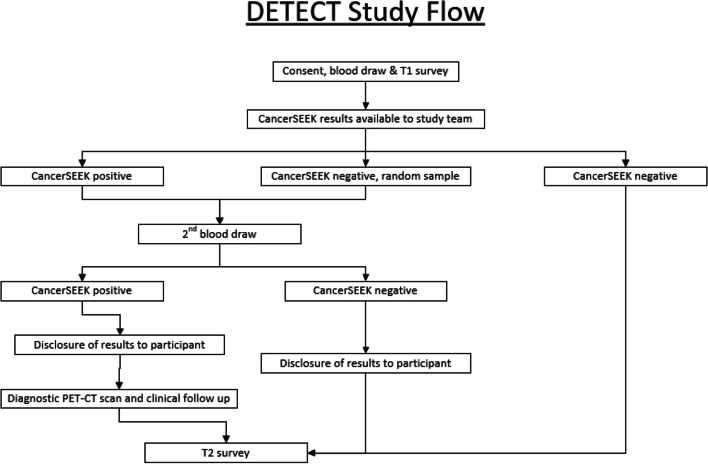
DETECT-A study design summary

**Fig. 2:**
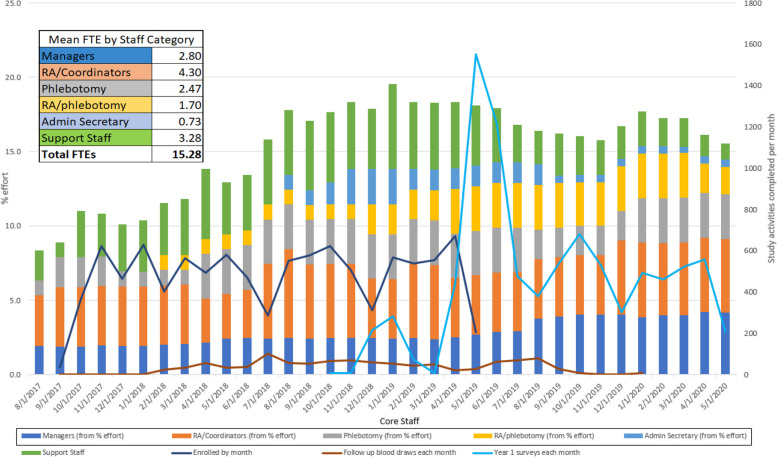
Enrollment and 1-year follow-up timeline showing staffing needs

DETECT-A evaluated the feasibility and safety of incorporating a multi-cancer early detection blood test into routine clinical care [11]. To analyze these outcomes, the study was designed to enroll 10,000 women, between the ages of 65–75, who had no prior history of cancer, in order to provide greater than 99% power to detect 20 or more cancers [11]. Recruitment began in August 2017 and enrollment was anticipated to be completed in 18 months. Study procedures are summarized in Fig. 1 and described in detail elsewhere [11]. Recruitment efforts, baseline enrollment visits, and follow-up activities overlapped chronologically, making it important to rapidly assess and adapt strategies to ensure that recruitment goals, protocol adherence, and patient satisfaction were simultaneously achieved (Fig. 2). Here we report the initial procedures for achieving the recruitment goal and methods for tracking processes and outcomes. Adaptations based on the results of process tracking are summarized in Results.

### Initial plan

The initial study team comprised two project managers, four research assistants, and one research phlebotomist, all of whom were fully funded by the study. Enrollment commenced at two locations where study staff had permanent office and/or clinic space. Initial recruitment methods included flyers, targeted mailings, and referrals from study staff and participants. Flyers that included a study phone number were displayed in Geisinger elevators and waiting areas in primary and specialty departments. Targeted mailings were sent in 101 batches (of fewer than 500 letters) to potentially eligible individuals who were identified by querying Geisinger’s electronic health record (EHR). Letters included return postcards and a phone number for recipients to express their interest or disinterest in participating. The letter explained that if we did not hear from them, we might call them in the future to assess their interest.

### Measures of implementation

Tracking and monitoring techniques to assess the recruitment progress, adherence to protocol procedures, and participants’ satisfaction are summarized below.

#### Recruitment procedures

**Fig. 3:**
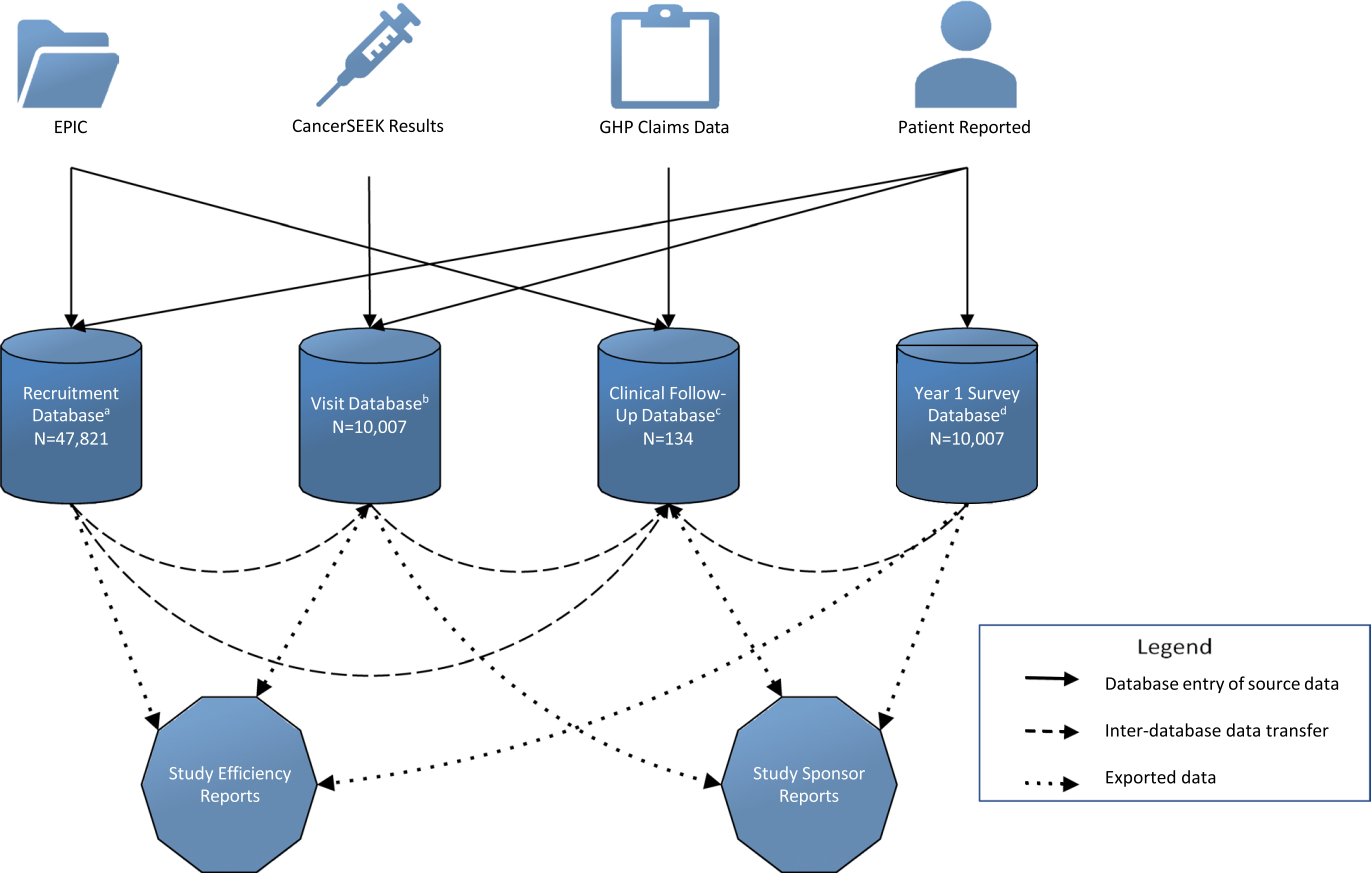
Outline of REDCap Databases, Dashboards and Reports. **a** The Recruitment Database was used to track recruitment activities for all potential participants that were sent a recruitment letter and anyone who responded to flyers, social media, or other recruitment methods. **b** The Visit Database was used to document enrolled participants’ CancerSEEK results and baseline visit data. **c** The Clinical Follow-up Database was used to collect follow-up data on those who completed a PET-CT scan, including cancer diagnoses. **d** The Year 1 Survey Database was used to collect Year 1 survey data, and to allow Geisinger’s Survey Research Core to administer surveys without needing training on the other databases

Due to the size of the cohort for this study, data were structured into several specialized Research Electronic Data Capture (REDCap) [13] databases. Access to these databases was given to staff based on their assigned study tasks (Fig. 3). The Recruitment Database was used to manage and monitor participant interest and progression throughout the study and provide weekly screening and enrollment metrics to the study collaborators. The study team used REDCap data to create Power BI dashboards to routinely assess important metrics such as the number of unfilled informed consent discussion appointment slots, and the recruitment and follow-up call assignments. If appointment slots were not being filled adequately, recruitment strategies and staff priorities were adjusted as needed (see Results). When potential participants were not interested in attending an enrollment event at currently available locations, we tracked their preferred location in REDCap and considered adding these locations as enrollment sites.

#### Protocol adherence procedures

Tracking and monitoring of protocol activities that occurred throughout the study were complex, requiring flexibility and coordination of study staff. Staff members were divided into ‘teams’ that had different priorities and tasks (Table 1). Each team monitored daily tasks and problem-solved in real time. Study activities were collected and tracked on paper, in the participants’ EHR, in the study REDCap databases, and in electronic files (Table 2). In addition to tracking and monitoring, the team was trained to identify potential issues proactively so that we could adapt to situations as needed. Protocol deviations and protected health information (PHI) breaches were handled immediately, documented in electronic spreadsheets and REDCap databases, and reported to the Geisinger’s institutional review board (IRB) at continuing review.

**Table 1:** Task prioritization by team

	**Visit Team**	**Office Team**
**Time Sensitive**	Staffing Sign-up Sheet	Managing Incoming Opt-in Postcards
	Visit Prep Checklist	Retrieving Voicemails (Communicating with Visit Team)
	Participant Check-in Process	Distribution of Outgoing Calls
	Participant Screening (Inclusion/Exclusion)	Incoming Calls (Communicating with Visit Team)
	Informed Consenting Process	Generating Appointment Sheets
	Specimen Collection and Documentation (Phlebotomy Form)	Reminder Calls for Upcoming Appointments
	Daily Specimen Manifest Entry	Finalizing and Printing Appointment Sheets
	Baseline Survey Administration	Daily Specimen Manifest Auditing (Real time)
	Giftcard Distrubution	Documenting Visits in EHR (Real time)
	Specimen Shipment	Sending Daily Specimen Manifest
**Time Flexible**	Baseline Survey Entry into REDCap	CancerSEEK Results Entry into REDCap
	Phlebotomy Form Entry into REDCap	Positive CancerSEEK Results Scanning into EHR
	Visit Paperwork Auditing	ICF Scanning into EHR Year 1 Survey Administration Survey Entry into REDCap Data Entry Giftcard Documentation Inventory and Ordering of Supplies Generating Barcodes for Specimen and Giftcard Tracking Creating Study Metrics Using Power BI Dashboards Financial Tracking and Reconciliation Filing Paper Source Documents Tracking and Addressing Participant Complaints Tracking Metrics for IRB Continuing Review Submission

**Table 2:** Data Collection and Tracking Mechanisms

Task	Paper	EHR	REDCap	Electronic File
Opt-in Postcards	S		C	
Staffing Sign-up Sheet				S
Visit Prep Checklist	S			
Check-in Documentation	S	C	C	
Screening (Inclusion/Exclusion)	S			C
Consent Documentation	S	C	C	C
Daily Specimen Manifest				S
Phlebotomy Documentation	S		C	
Baseline Survey	S		C	C
Gift Card Distribution	S			C
Incoming and Outgoing Call Documentation			S	C
Appointment Tracker	C	C	C	S
CancerSEEK Results		C	C	S
Annual Follow-up Surveys	S		S	C
Medical History Data for CancerSEEK Positives			C	S
Inventory and Ordering	C			C
Specimen Barcodes and Gift Cards	C			S
Study Metrics Using Power BI Dashboards				S
Participant Complaints			S	C
Metrics for IRB Continuing Review Submissions				S

S = source documentation in which data collected was first recorded

C = copy of data documentation that was later entered into an electronic data capture system

#### Participant experience procedures

Throughout the study, any concerns relating to a visit location were reported to a study coordinator. Participants were also encouraged to contact the study’s genetic counselors for questions about their results, and the study team or Geisinger’s IRB with any general problems, questions, or concerns.

## Results

The DETECT-A study enrolled 10,007 participants over the course of 22 months, which was four months longer than our anticipated timeline. Table 3 shows a monthly breakdown of adaptations to the recruitment methods and locations used to achieve the enrollment goal. Adaptations to our recruitment, enrollment, protocol, and participant experience processes are summarized in Table 4. All adaptations required submission to the IRB as amendments and were approved accordingly. A common theme that emerged was the need to increase participant-centered flexibility in recruitment and enrollment procedures. A variety of methods were used for participants to express interest in participation, including by returning a postcard, calling the study team, or completing an eligibility questionnaire online. Visit locations were expanded to 22 sites from which the participants could choose. During recruitment visits participants could also choose to be a part of a group or an individual consenting session.

**Table 3:** DETECT-A Recruitment Timeline

	Recruitment Methods							Cumulative Enrollment Metrics			
Month of Recruitment (Month # and abbreviation)	Mass Mailings (M)^1^	Referrals (R)^2^	Flyers (F)^3^	Social Media (S)	News Media (NM)	Newsletters (N)	Community Outreach Events (C)	Locations Used	Locations Available	Enrolled per Month	Enrolled
M1 Aug	M^1^	R^1^	F^1^					0	0	0	0
M2 Sep	M^1^	R^1^	F^1^				C	2	2	36	36
M3 Oct	M^1^	R^1^	F^1^	S	NM			4	4	364	400
M4 Nov	M^1^	R^1^	F^1^	S	NM			3	4	621	1021
M5 Dec	M^1^	R^1^	F^1^	S			C	4	4	464	1485
M6 Jan	M^1^	R^1^	F^1^					4	5	629	2114
M7 Feb		R^1^	F^1^	S				5	5	403	2517
M8 Mar		R^1^	F^2^			N		7	7	564	3081
M9 Apr		R^1^	F^**2**^		NM			8	8	491	3572
M10 May		R^1^	F^2^	S				9	14	581	4153
M11 Jun		R^1^	F^2^	S				14	18	470	4623
M12 Jul		R^1^	F^2^					10	18	284	4907
M13 Aug		R^1^	F^2^		NM	N		14	18	552	5459
M14 Sep	M^2^	R^1^	F^2^	S			C	15	18	577	6036
M15 Oct	M^2^	R^1^	F^2^	S		N	C	16	20	666	6702
M16 Nov	M^**3**^	R^1^	F^2^	S		N	C	16	21	462	7164
M17 Dec	M^3^	R^2^	F^2^	S		N	C	15	21	310	7474
M18 Jan	M^3^	R^2^	F^2^	S	NM	N		15	22	566	8040
M19 Feb	M^3^	R^2^	F^2^			N		15	22	537	8577
M20 Mar	M^3^	R^2^	F^2^			N		16	22	555	9132
M21 Apr		R^2^	F^2^			N		16	22	670	9803
M22 May		R^2^	F^2^			N		14	22	204	10,007

^1^Initial mass mailings (M^1^) included an invitation letter and a return postcard. At month 14, the return postcard was replaced by an information card (M^2^) that featured a photo of study participants. At month 16, the details from both the information card and invitation letter were incorporated into a single invitation card (M^3^)

^2^Initial referrals (R^1^) were accepted from physicians, research staff, and participants. At month 17, we added a Refer-A-Friend program (R^2^) until enrollment ended

^3^Initial flyers (F^1^) included only text and were printed by study staff. At month 8, Geisinger’s Marketing and Communications department designed flyers (F^2^) with stock photos and tear-off contact information

**Table 4:** Rapid Cycle Evaluation of Study Implementation

	Problems	Consequences	Solutions
**Recruitment**	1. Slow response time from recruitment materials 2. Labor intensive 3. Underwhelming graphic design in recruitment materials 4. Recruitment limited to Geisinger patient population	1. Patients aged out of eligibility 1. Unused consenting visit slots 2. Less staff availability for visits 3. Recipients doubted authenticity of materials 3. Recruitment materials easy to overlook 4. Depleted pool of potentially eligible participants	1. Started making recruitment calls before postcards were returned 1. Removed return postcard from mailings 2. Outsourced printing and mailing 2. Outsourced non-responder recruitment calls 3. Utilized Geisinger’s Marketing & Communications department for ads 4. Placed flyers in the community 4. Added advertisements (social media, news media, newsletters) 4. Added referrals from participants 4. Added community outreach events
**Enrollment**	1. Staff time was divided between recruitment and enrollment and other study activities 2. Inefficient informed consenting process 3. Interested participants unwilling/unable to travel to available locations	1. Fluctuating monthly enrollment totals 2. Individual sessions limited number of participants enrolled per day 2. No-shows and cancellations impacted the number of participants enrolled per day 2. Consenter fatigue 3. Interested participants could not be scheduled for enrollment visit	1. Hired, cross-trained, and utilized staff from other departments 2. Group consenting sessions 2. Accounted for no-shows and cancellations by opening additional appointment slots 3. Secured additional enrollment locations for participant convenience and contacted particpants again
**Protocol and Procedure Adherence**	1. A staff member formatted an appointment sheet incorrectly 2. Photocopier not available at most visit sites required participants to sign two copies of the ICF 3. Paperwork (inclusion form, ICF, gift card confirmation, phlebotomy form, and baseline survey) was challenging to keep organized at visits	**Protocol deviations:** 1. Printed appointment information was inaccurate, and participants completed wrong visit types (one paricipant was consented twice and one participant had a blood draw before signing consent) 2. Participants only signed onecopy of ICF and took it home, which resulted in missing paperwork **PHI breach:** 3. In two instances, participants took other participants paperwork home requiring reports to IRB and Privacy Office	1. Staff was trained on how to correctly format appointment sheets 1. Assigned appointment sheet formatting to a specific staff member 2. Implemented audits checking for unsigned or missing ICFs during visits 3. Utilized folders for each participant to organize paperwork at visits
**Participant Experience**	1. Inconsistent turnaround time for returning PET-CT scan results 2. Utilized 22 different enrollment sites, each with unique challenges	1. Five participants complained 1. Participants reported stress or anxiety when calling to inquire about PET-CT scan results 2. Site limitations included: a. Poor parking b. Lack of privacy c. Lack of signage to visit area d. Lack of operational management at non-clinic sites	1. Added primary care investigators to the study to return non-cancerous PET-CT results, requiring study oncologists to only return suspicious findings 2. Problem locations were no longer used, or processes were modified a. Detailed parking instructions were provided during scheduling and reminder calls b. Altered visit setup to accommodate for more privacy and safety for participants c. Designed portable study signage for use at all sites d. Assumed more responsibility at sites that lacked operational management

### Recruitment procedures – problems and consequences

We identified several challenges in the initial recruitment method used in the first two months (Table 4). The time it took for potential participants to return the postcards indicating they were interested resulted in some of them no longer meeting eligibility criteria, which impacted our ability to fill appointment times at study visits. Management of the recruitment mailing process and return postcards was also labor intensive for the staff, which limited their availability for visits. The graphic design of the initial recruitment materials (i.e., invitation letters and return postcards) were bland and did not elicit interest from recipients who doubted their authenticity. Mailings were limited to Geisinger patients, which resulted in a smaller pool of potentially eligible participants early in the study. Finally, mailings and flyers alone did not generate a sufficient number of interested participants in a timely manner to meet weekly enrollment targets.

### Recruitment procedures–solutions

**Fig. 4:**
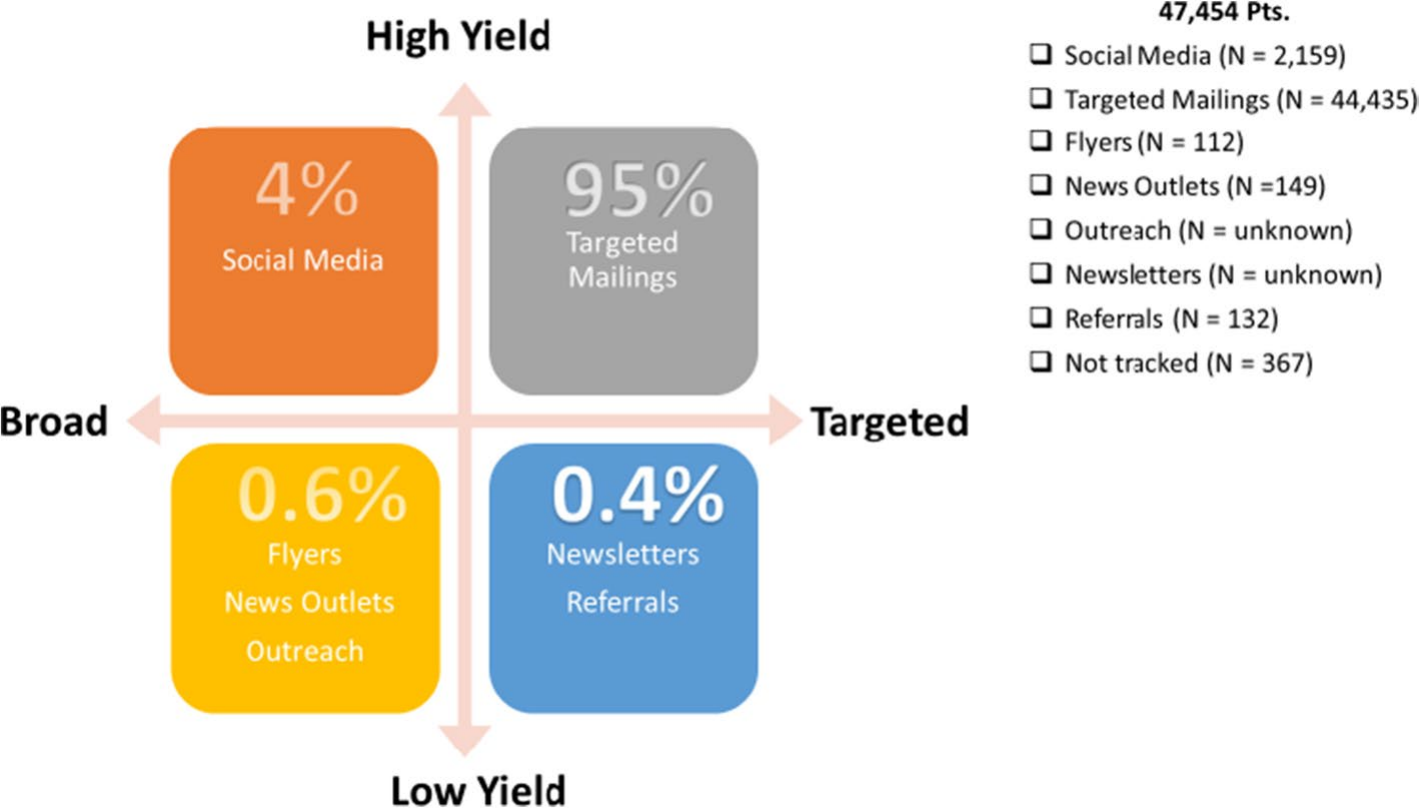
Percent yield. Percentages shown above represent the number of participants who enrolled in the study by recruitment method category. Targeted recruitment methods focused on individuals who were more likely to meet inclusion/exclusion criteria. Broad methods reached a larger population that resulted in a lower percentage of eligible individuals. Mass mailings and social media advertisements resulted in the highest yield of interested and eligible individuals

To reduce response turnaround time from the initial recruitment methods, modifications were made to the targeted mailing process and to the flyers. The printing and the mailing of recruitment materials were both outsourced to Geisinger’s Digital Print and Mail Center. Geisinger’s Marketing & Communications department created recruitment content with more engaging graphic design. Postcards were eliminated to make the recruitment process more time efficient. We also supplemented our recruitment efforts with methods designed to reach non-Geisinger patients, including advertisements (social media, news media, newsletters, flyers), referrals from participants, and community outreach (Table 4). Both targeted and broad recruitment methods were used in tandem, as necessary solutions to meet weekly recruitment targets (Fig. 4).

Additionally, Facebook advertisements were created using demographic and geographic parameters. Readers who clicked the ad were routed to the DETECT-A website, where they were provided with contact information for the study team and given the opportunity to complete an eligibility questionnaire. The study team reviewed the eligibility of each submission, including EHR review for Geisinger patients. An email was sent to individuals if they were not eligible. Individuals who were potentially eligible received a phone call where inclusion/exclusion criteria were confirmed. Social media recruitment, which had a link to opt-in electronically that directly alerted the study staff, had the quickest response time from potential participants compared to other recruitment methods. At the end of the study, mass mailings had the highest yield of interested and eligible individuals accounting for 95% of those who enrolled in the study. Social media advertisements followed with 4% (Fig. 4).

To broaden our reach of potential study candidates, study staff participated in multiple community outreach events where the target population was represented, such as local community fairs, health and wellness fairs, and community luncheons for older individuals. Stories about the DETECT-A study and its investigators were featured in local newspapers, on Geisinger-sponsored televised health segments, and on local news programs. Upcoming enrollment events were highlighted in standing communications from Geisinger to members of the Geisinger Health Plan (GHP) [14], Silver Circle [15], and other large Geisinger research projects like the MyCode Community Health Initiative [16].

Study staff placed the flyers in new locations, including wellness centers, senior centers, churches, and local businesses. Currently enrolled DETECT-A participants were encouraged to share study flyers with other potential participants. Since this approach was successful, we developed a “Refer-a-Friend" mailing campaign for the last 6 months of enrollment (see Table 3). Participants who referred eligible friends and family during a designated time frame were entered into a one-time gift card drawing. All remaining recruitment methods mentioned above contributed to 1% of those who enrolled in the study (Fig. 4).

### Enrollment procedures–problems and consequences

Enrolling individuals presented additional challenges beyond those encountered during recruitment. Interested individuals were sometimes unwilling or unable to travel to available enrollment locations to provide informed consent, negatively impacting enrollment numbers. Individual informed consent sessions resulted in consenter fatigue, a lower daily capacity for enrollment, and a loss in productivity when potential participants canceled or did not show for scheduled appointments. Recruitment efforts and enrollment events competed for staff time, which was reflected in fluctuating enrollment numbers from month to month. Months with heavy enrollment meant staff were largely deployed to visits, which were then followed by months with unfilled appointment slots as staff had less time to devote to recruitment calls (Fig. 2).

### Enrollment procedures–solutions

We organized two teams to meet recruitment, enrollment, and other study activity demands (Table 1). The Visit and Office Teams’ tasks were prioritized in categories of time sensitive versus time flexible. Visit logistics were designed to allow for efficiency when the Visit Team became mobile. Staff traveled in study vehicles to the enrollment locations with tables, chairs, lab kits, phlebotomy supplies (including snacks and water for participants post blood draw), signage, and paperwork. Over the 22 months of recruitment, locations were expanded to a total of 22 sites across the Geisinger service area based on feedback from recruitment calls (Table 3). These sites included Geisinger and non-Geisinger spaces. The Geisinger spaces consisted of exam rooms, conference rooms, multipurpose rooms, employee breakrooms, community rooms, and the Geisinger Commonwealth School of Medicine. Non-Geisinger spaces included a community center, an athletic facility, and an indoor courtyard.

Hourly scheduled group consenting sessions were instituted. The size and the number of groups were determined by site layout and staffing. The ideal layout was a large open room where participants could move through stations that were staffed to conduct informed consent sessions, surveys, and blood draws. Maintaining flexibility in staffing meant that one consenter could meet with up to 15 participants at once or multiple consenters could meet with smaller groups, all while continuing to accommodate requests for individual consenting. Post-consent blood draws and survey completion could occur in either order depending on the size of the group. Overbooking appointments lessened the impact of cancelations and no-shows. Late participants were provided a copy of consent and asked to wait for the next scheduled session. The group consenting model allowed for adequate staff rest breaks between sessions and increased the number of consented participants per day by 2–5 times (up to 103 participants per day).

We cross-trained fully funded staff and employed research support department staff to assist with recruitment phone calls, conducting informed consent, and blood draws, which freed up fully funded staff for office tasks (Fig. 2). We utilized Geisinger’s Survey Research Core to assist with recruitment calls, scheduling, and survey administration, allowing us to expand these activities to nights and weekends. We were able to leverage additional system-wide internal resources, such as having Research Assistants train at Geisinger’s School of Phlebotomy to perform venipunctures, which expanded our team of research phlebotomists and improved visit efficiency. Genetic counselors and clinicians were part of a Multidisciplinary Review Committee for the study, who reviewed CancerSEEK positive results and recommended next steps.

Effort was tracked by Project Managers who viewed and reconciled monthly expense and effort reports provided by Research Finance. Genetic counselors and clinicians who were Sub-Investigators on the study billed time and effort to the study. The clinicians who saw participants after a PET-CT scan charged their time as a level of service for that visit, not through time and effort. Level of service costs were billed to the study, not to the participant or their insurance.

In total, 111 employees billed their time and effort to the study during the 22 months of recruitment and the first 12-months of follow-up, which translated to an average of 15.28 full-time equivalent (FTE) during recruitment and follow-up. This included managers at 2.80 FTE, research assistants and coordinators at 4.30 FTE, phlebotomists at 2.47 FTE, cross trained phlebotomists at 1.70 FTE, administrative secretaries at 0.73 FTE and support staff at 3.28 FTE. These diverse staffing resources allowed for division of labor by area of expertise. Additionally, our core staff was cross trained for both office and visit tasks (Table 1), which gave us the flexibility to adapt procedures in real time as the study demanded.

### Protocol adherence procedures–problems and consequences

We had four types of protocol deviations: using outdated informed consent (ICF) versions, enrolling more participants than were IRB-approved, misplacing original ICFs, and signing an ICF after completing venipuncture. There were two types of PHI breaches reported to the privacy office, including a mix-up of paperwork at the visits, and mailing paperwork to the wrong participant. There were two issues of non-compliance for unauthorized disclosure of health information for internally providing a patient list that included PHI to a statistician who was not yet added to IRB study application and accidental sharing of unapproved PHI on an internal and private group communication board. Table 4 details the problems that resulted in adaptations to our processes. It was determined the problems and consequences were largely related to the use of paper source documents, which were inefficient, time-consuming, and vulnerable to human errors.

### Protocol adherence procedures–solutions

#### Protocol deviations

To avoid future protocol deviations, it was necessary to periodically retrain staff on certain processes. For complex and technical tasks, we designated a point person to have primary responsibility to decrease protocol errors. For example, an assigned staff member exported reports to generate appointment sheets, which led to better organization and easier identification of participants’ visit types at each visit. Multiple staff were retrained as backups and were used when needed on that assignment. Identification of visit types were key because participants were at different stages of the study. For instance, a baseline visit included the informed consent process, completion of a study developed questionnaire (Supplemental Baseline Questionnaire 1), and a blood draw versus follow up blood draw or redraw appointments for those participants who were already consented into the study.

We implemented checks throughout large visit days to ensure all ICFs were signed and filed appropriately, which enabled us to identify missing signatures or forms and obtain them before the participant left the visit.

#### PHI breaches

To facilitate adherence to the protocol procedures, each participant was given a folder at the start of the visit containing the inclusion/exclusion verification sheet, gift card confirmation, and phlebotomy form, as well as the ICF and baseline survey. The participant kept the folder with them throughout the visit. At the conclusion of their visit, study staff retrieved original paperwork from the folder and returned copies of the ICF and gift card confirmation form to the participants.

### Participant experience procedures–problems and consequences

Four of the 22 enrollment sites presented challenges that were identified by participants using a patient-satisfaction survey. Dissatisfaction included limited parking, privacy concerns, lack of signage directing participants to visit areas, and limited facility oversight at non-clinic sites. Of clinical importance, five participants who underwent a PET-CT scan reported anxiety due to prolonged wait times between imaging and result disclosure. Also, there were complaints related to study design. For example, three participants that had a negative CancerSEEK test were upset they did not hear from the team again until it was time to complete their Year 1 survey. We also had three participants who were upset that they were diagnosed with cancer despite their CancerSEEK test being negative.

### Participant experience procedures–solutions

We discontinued use of enrollment sites that prompted multiple participant complaints. To improve participant experience at remaining sites, we altered visit set-ups, provided more detailed parking instructions, and displayed portable study signage. It was also necessary for the core study staff to take more responsibility at sites that lacked operational management.

We increased the number of clinician investigators on the study to assist with the timely return of negative and positive PET-CT scan results. Study staff triaged the findings to the appropriate study clinicians. Specifically, primary care physicians on the study were enlisted to return imaging findings that were not concerning for cancer. This allowed study oncologists to focus on only returning the findings suggestive of cancer. In all instances, participants’ primary care physicians were also notified of the results and recommended next steps via EHR message, fax, or phone call. We did not address the study design concerns participants raised but we did offer genetic counseling visits for those who were upset by their result, a new cancer diagnosis, or wanted further information.

## Discussion

The effective and efficient recruitment of large numbers of individuals over short time periods is critical to translating research findings into practice [1, 2, 12]. The lessons learned from the successful recruitment into a large, prospective cohort study, DETECT-A, can inform future large recruitment efforts and foreshadow important clinician- or patient-level implementation obstacles to be anticipated. Our findings in a rural, aging population highlight successful recruitment strategies and the importance of real-time data-driven adaptations to these strategies. These results, which are consistent with recent recruitment literature [17–20], demonstrate the importance of iterative adaptation to a priori recruitment and enrollment strategies based on timely evaluation of available data as key to our study’s ability to meet enrollment targets [20]. Using existing data capture systems like REDCap [13] can streamline the ease with which data from these recruitment strategies can be analyzed.

The most impactful adaptations to recruitment strategies identified were related to group consenting, staffing, and participant experience. These solutions may be effective within other study designs and organizational contexts. A variety of methods were used for participants to express interest in participation, including by returning a postcard, calling the study team, or completing an eligibility questionnaire online. Initially, the study used individual consenting sessions, as is typical in many studies. It was quickly apparent from enrollment figures that this approach was inefficient and recruitment goals would not be reached in the necessary timeframe. Shifting to a group consenting format resulted in efficiencies that facilitated the ability to reach goals on target without negatively impacting adherence to study protocol. Anecdotally, we observed that group consenting enhanced conversational dynamics and allowed for deeper and more meaningful discussion of the informed consent form, an experience consistent with the impact of group dynamics in clinical settings [21–24]. We recommend using a variety of strategies, particularly when attempting to overcome recruitment barriers among populations underrepresented in research [19]. For example, even with the increase in popularity of e-consenting and remote visits, studies seeking to enroll elderly participants may choose to maintain in-person consenting visits as an option for individuals who are less comfortable with computers or do not have access to a computer or device. Studies involving a blood draw or other procedures would still require an in-person interaction thus using multiple strategies (e.g., offering informed consent by telephone, chatbot, telehealth, and in-person) offers opportunities to be as inclusive as possible and limit selection bias.

Our experience also underscores challenges of, and potential solutions to, recruiting in rural settings. Geisinger’s service area is predominantly rural, and covers a large geographic area, which sometimes required participants to travel long distances to reach an enrollment location. Rural populations can and should have the opportunity to participate in research studies in spite of geographic distance; our effort to utilize multiple enrollment spaces was successful in attracting these participants. We selected local Geisinger clinics and trusted community spaces that were familiar to our population, such as community centers and athletic facilities. Our expanding recruitment efforts and study activities required a rapid increase in study staff, and this increased staff size allowed us to expand to many of the requested locations that were more convenient for participants. Some of these locations were in less populous areas, so by scheduling at more than one site per day we were able to use staff time more efficiently while meeting daily enrollment targets. The study leased two vehicles for staff travel to cover the additional visit sites.

Another important lesson was to not undertake tasks for which other groups are experts. For example, we utilized resources at Geisinger such as Marketing and Communications and the Digital Print and Mail Center in the design of posters and other recruitment materials. In addition, we found that adding small efforts from several staff members outside the core study team can meaningfully supplement the team’s capacity to focus on participant-centered enrollment. For interventional trials, integrating the disclosure of study results easily and seamlessly into routine clinical care without adding unfunded work for clinicians is key [18]. We did this by triaging the findings to the appropriate study clinicians, allowing study oncologists to focus on only returning the findings suggestive of cancer. We also notified the participants’ primary care physicians of the results and recommended next steps via EHR message, fax, or phone call.

When it comes to capturing and tracking data and source documents, we recommend electronic capture mechanisms whenever possible. Robust electronic tracking of study processes allows the team to identify ineffective processes, adapt quickly based on data, and avoid protocol deviations. It is also critical to link multiple databases when applicable. We initially thought that having a separate REDCap database for each subset of participants as they progressed through the study would make data management easier and quicker. However, we recommend using a single database whenever possible, as the administrative burden of transferring essential data between databases, and updating critical fields in multiple places, was significant, and prone to data entry errors.

Though we did not intend to do so from the outset, we used several of the implementation strategies described by Powell et al., including changing service sites, developing and implementing tools for quality monitoring, and promoting adaptability [25]. As we adapted recruitment strategies throughout the study, we realized, as others have, the importance of considering implementation strategies and associated outcomes during study planning [17]. That approach could have allowed us to focus on assessing racial and ethnic representation in the DETECT-A cohort and implementing strategies to improve diversity. Such strategies would include translating all recruitment and patient-facing study materials into multiple languages and training research staff on the many medical interpreter resources used by clinic staff to effectively communicate in patients’ preferred languages. The importance of this missed opportunity is highlighted by a post-hoc comparison that found the DETECT-A cohort to be significantly less racially and ethnically diverse (Chi-square = 420.45, *p* < 0.001 and Chi-square = 1001.86, *p* < 0.001, respectively) than the overall female Geisinger population of the same age range. As noted by Swanton et al., it could be useful to track the “number needed to invite”—the number of invitations that need to be sent to achieve one person enrolled into a study – and determine whether this number differs between groups [4]. We missed an opportunity to collect race and ethnicity of all invited individuals during our recruitment phase, preventing us from comparing recruitment rates by race or ethnicity. Collecting age, gender, race, ethnicity, geographical area, and socioeconomic variables during recruitment could be beneficial to calculate the number needed to invite among various categories. Evidence from other cancer screening studies has shown that robust, sustained engagement in underrepresented communities and shared decision making can encourage enrollment of diverse cohorts [26]. Identifying and understanding the distinct barriers of diverse populations is an important step to improving outreach and communication with people. Allocating resources and costs to fully engage those underserved populations is key.

Based on our experiences in the DETECT-A study, our key findings can be summarized by these five recommendations to consider from the outset of a clinical research study: 1. offering multiple recruitment and consenting options, especially with older participants, as they may be less comfortable with using or don’t have access to a computer or device and prefer in-person visits 2. offering research visits at many clinics and trusted community organizations in rural areas that are frequently visited, such as community centers and athletic facilities 3. identifying and utilizing available resources and teams so that the core study team could focus on participant-centered enrollment, and for interventional trials integrating the disclosure of study results easily and seamlessly into routine clinical care without adding unfunded work for clinicians 4. capturing and tracking data and source documents electronically as much as possible and finally 5. using an implementation science framework, adaptation tracking, and various implementation strategies to address needs from the beginning of the study. In closing, including metrics aligned with the measurement of adaptations, such as who instituted what, when, and why, and using any of the frameworks as described in implementation science would improve post-hoc analysis of the impact and the value of individual strategies and allow for further transparency in value trade-offs to improve generalizability to other contexts [27–31]. By acknowledging these limitations, we hope that the post-hoc analysis of implementation outcomes described in this manuscript will be valuable for informing future measurements and tracking adaptations.

## Conclusion

Recruiting rapidly for large prospective cohort studies – and thereby facilitating clinical translation – requires a nimble, creative approach that marshals available resources and changes course according to data. Planning a rigorous assessment of a study’s implementation outcomes prior to study recruitment can support recruitment of a diverse and representative cohort and facilitate translation into practice. This can be accomplished by proactively and continuously assessing and revising implementation strategies.

### Supplementary Information

**Supplementary Material 1.**

## Data Availability

All data generated and/or analyzed during this study are included in this published article and its supplementary information files.

## Abbreviations

MCED: Multi-cancer Early Detection
EHR: Electronic Health Record
REDCap: Research Electronic Data Capture
PHI: Protected Health Information
IRB: Institutional Review Board
GHP: Geisinger Health Plan
FTE: Full-time equivalent
ICF: Informed Consent Form

## References

[1] Liu MC, Oxnard GR, Klein EA (2021). Sensitive and specific multi-cancer detection and localization using methylation signatures in cell-free DNA. Ann Oncol.

[2] The All of Us Research Program Investigators. The “All of Us” research program. N Engl J Med. 2019;381:668–76. 10.1056/NEJMsr1809937 PMC8291101 31412182

[3] Schrag D, Beer TM, McDonnell CH 3rd, Nadauld L, Dilaveri CA, Reid R, Marinac CR, Chung KC, Lopatin M, Fung ET, Klein EA. Blood-based tests for multicancer early detection (PATHFINDER): a prospective cohort study. Lancet. 2023;402(10409):1251–60. 10.1016/S0140-6736(23)01700-2. 10.1016/S0140-6736(23)01700-2 PMC11027492 37805216

[4] Swanton C, et al. NHS-Galleri Trial Design: Equitable study recruitment tactics for targeted population-level screening with a multi-cancer early detection (MCED) test. JCO. 2022;40:TPS6606–TPS6606. 10.1200/JCO.2022.40.16_suppl.TPS6606.

[5] Carey DJ, Fetterolf SN, Davis FD (2017). The geisinger MyCode community health initiative: an electronic health record–linked biobank for precision medicine research. Genet Med.

[6] Ramirez AH, Sulieman L, Schlueter DJ, et al. The all of us research program: Data quality, utility, and diversity. Patterns. 2022;3(8). 10.1016/j.patter.2022.100570. 10.1016/j.patter.2022.100570 PMC9403360 36033590

[7] Nipp RD, Hong K, Paskett ED. Overcoming Barriers to Clinical Trial Enrollment. American Society of Clinical Oncology educational book. American Society of Clinical Oncology. Annual Meeting. 2019;39:105–14. 10.1200/EDBK_243729. 10.1200/EDBK_243729 31099636

[8] Forsat ND, Palmowski A, Palmowski Y, Boers M, Buttgereit F (2020). Recruitment and retention of older people in clinical research: a systematic literature review. J Am Geriatr Soc.

[9] Kim NH, Wilson N, Mashburn T, et al. Lessons learned recruiting a diverse sample of rural study participants during the COVID-19 pandemic. Int J Drug Policy. 2021;97. 10.1016/j.drugpo.2021.103344. 10.1016/j.drugpo.2021.103344 PMC8556070 34186474

[10] Greer TL, Walker R, Rethorst CD (2020). Identifying and responding to trial implementation challenges during multisite clinical trials. J Subst Abuse Treat.

[11] Lennon AM, et al. Feasibility of blood testing combined with PET-CT to screen for cancer and guide intervention. Science. 2020;369:eabb9601. 10.1126/science.abb9601. 10.1126/science.abb9601 PMC7509949 32345712

[12] Berger PB, Henry Y, Harkins V, Ferrari A. Use of an electronic health record to optimize site performance in randomized clinical trials. J Clin Trials. 2015;05(01). 10.4172/2167-0870.1000208.

[13] Harris PA, Taylor R, Thielke R, Payne J, Gonzalez N, Conde JG (2010). Research electronic data capture (REDCap)—A metadata-driven methodology and workflow process for providing translational research informatics support. J Biomed Inform.

[14] Geisinger health plan. https://www.geisinger.org/health-plan. Updated 2024. Accessed 11 Feb 2022.

[15] Silver circle. https://www.geisinger.org/health-and-wellness/silver-circle. Updated 2024. Accessed 11 Feb 2022.

[16] MyCode community health initiative. https://www.geisinger.edu/gchs/research/mycode. Updated 2024. Accessed 11 Feb 2022.

[17] Ahonkhai AA, Wudil UJ, Dankishiya FS (2021). Strategies for successful clinical trial recruitment of people living with HIV in low- and middle-income countries: lessons learned and implementation implications from the Nigeria renal risk reduction (R3) trial. Curr HIV/AIDS Rep.

[18] Taft T, Weir C, Kramer H, Facelli JC (2019). Primary care perspectives on implementation of clinical trial recruitment. J Clin Trans Sci.

[19] Masese RV, Demartino T, Bonnabeau E (2020). Effective recruitment strategies for a sickle cell patient registry across sites from the sickle cell disease implementation consortium (SCDIC). J Immigrant Minority Health.

[20] Huang B, De Vore D, Chirinos C, et al. Strategies for recruitment and retention of underrepresented populations with chronic obstructive pulmonary disease for a clinical trial. BMC Med Res Methodol. 2019;19(1). 10.1186/s12874-019-0679-y. 10.1186/s12874-019-0679-y PMC6385381 30791871

[21] Lieberman MA, Golant M, Altman T (2004). Therapeutic norms and patient benefit: cancer patients in professionally directed support groups. Group Dyn Theory Res Pract.

[22] Shaw ME (1981). Group dynamics: The psychology of small group behavior.

[23] Wilson SR (1997). Individual versus group education: Is one better?. Patient Educ Couns..

[24] Shechtman Z, Toren Z (2010). The association of personal, process, and outcome variables in group counseling: testing an exploratory model. Group Dyn Theory Res Pract.

[25] Powell BJ, Waltz TJ, Chinman MJ, et al. A refined compilation of implementation strategies: Results from the expert recommendations for implementing change (ERIC) project. Implementation Sci. 2015;10(1). 10.1186/s13012-015-0209-1. 10.1186/s13012-015-0209-1 PMC4328074 25889199

[26] Thompson C, Buchanan A, Myers R, Weinberg DS. Integrating primary care, shared decision making, and community engagement to facilitate equitable access to multi-cancer detection clinical trials. Front Oncol: Cancer Epidemiol Prev. 2023;13. 10.3389/fonc.2023.1307459. 10.3389/fonc.2023.1307459 PMC10937460 38486933

[27] Glasgow RE, Battaglia C, Mccreight M, Ayele RA, Rabin BA. Making implementation science more rapid: Use of the RE-AIM framework for mid-course adaptations across five health services research projects in the veterans health administration. Front Public Health. 2020;8. 10.3389/fpubh.2020.00194. 10.3389/fpubh.2020.00194 PMC7266866 32528921

[28] Treichler EBH, Mercado R, Oakes D, et al. Using a stakeholder-engaged, iterative, and systematic approach to adapting collaborative decision skills training for implementation in VA psychosocial rehabilitation and recovery centers. BMC Health Serv Res. 2022;22(1). 10.1186/s12913-022-08833-2. 10.1186/s12913-022-08833-2 PMC9759039 36528579

[29] Mccarthy MS, Ujano-De Motta LL, Nunnery MA, et al. Understanding adaptations in the veteran health administration’s transitions nurse program: Refining methodology and pragmatic implications for scale-up. Implementation Sci. 2021;16(1). 10.1186/s13012-021-01126-y. 10.1186/s13012-021-01126-y PMC8276503 34256763

[30] Moullin JC, Nevedal A, Mccreight M, et al. Using a longitudinal multi-method approach to document, assess, and understand adaptations in the veterans health administration advanced care coordination program. 10.3389/frhs.2022.970409 PMC10012685 36925896

[31] Smith JD, Norton WE, Mitchell SA, et al. The longitudinal implementation strategy tracking system (LISTS): Feasibility, usability, and pilot testing of a novel method. Implement Sci Commun. 2023;4(1). 10.1186/s43058-023-00529-w. 10.1186/s43058-023-00529-w PMC10683230 38017582

