# Supplementary material for: Real-time evaluation and adaptation to facilitate rapid recruitment in a large, prospective cohort study
Source: BMC Health Serv Res. 2024 Mar 13;24:336. doi: 10.1186/s12913-024-10750-5 (PMC10938733; doi:10.1186/s12913-024-10750-5)
Supplement: Supplementary file 1 — Supplementary Material 1. [file 12913_2024_10750_MOESM1_ESM.pdf]

<u>Demographics</u>:

1. Have you ever been diagnosed with cancer?
   a. Yes
      - If yes, check all that apply:
         - ☐ None
         - ☐ Breast Cancer, Age at Diagnosis _____
         - ☐ Colon Cancer, Age at Diagnosis _____
         - ☐ Liver Cancer, Age at Diagnosis _____
         - ☐ Ovarian Cancer, Age at Diagnosis _____
         - ☐ Esophageal Cancer, Age at Diagnosis _____
         - ☐ Pancreatic Cancer, Age at Diagnosis _____
         - ☐ Stomach Cancer, Age at Diagnosis_______
         - ☐ Other, Specify Site_______________ Age at Diagnosis___
   b. No

2. What is your current age? _______

3. What is your current height? ______Feet ______Inches

4. What is your current weight? ______Pounds

5. Has your weight varied in the past 12 months?
- ☐ Remained stable
- ☐ Intentionally gone up more than 10 pounds
- ☐ Unintentionally gone up more than 10 pounds
- ☐ Intentionally gone down more than 10 pounds
- ☐ Unintentionally gone down more than 10 pounds

6. Do you consider yourself Hispanic or Latino?
- ☐ Yes
- ☐ No
- ☐ Don't know
- ☐ Prefer not to answer

7. What race or races do you consider yourself to be? (check all that apply)

- ☐ American Indian or Alaska Native
- ☐ Asian
- ☐ Black or African American
- ☐ Native Hawaiian or Pacific Islander
- ☐ White
- ☐ Other
- ☐ Don’t know
- ☐ Prefer not to answer

8. Are you of Ashkenazi Jewish descent?

- ☐ Yes
  - If yes, please check the number of grandparents who were Ashkenazi Jewish
    - ☐ 1
    - ☐ 2
    - ☐ 3
    - ☐ 4 or more
- ☐ No
- ☐ Don’t know
- ☐ Prefer not to answer

9. Which is the highest grade or level of education you have completed?

- ☐ 8th grade or less
- ☐ Some high school
- ☐ High school graduate or GED
- ☐ Vocational, technical or business school
- ☐ Some college or Associates Degree
- ☐ Four-year college graduate
- ☐ Graduate or professional school
- ☐ Other

<u>Smoking History</u>:

1. Have you smoked at least 100 cigarettes in your entire life?
- ☐ Yes
- ☐ No
  - If not, skip to Question #6
- ☐ Don't know
- ☐ Prefer not to answer

2. Do you currently smoke cigarettes every day, some days, or not at all?
- ☐ Every day
- ☐ Some days
- ☐ Not at all
  - If no, skip to Question #4
- ☐ Don't know
- ☐ Prefer not to answer

3. At what age did you start smoking? _________

4. If you no longer smoke cigarettes regularly, at what age did you stop? _______

5. When you smoked, on average how many cigarettes did you/do you smoke per day?
- ☐ Less than a half a pack (1-10)
- ☐ Less than a pack (10-20)
- ☐ A pack a day
- ☐ 1-2 packs a day
- ☐ More than 2 packs a day

<u>Only answer the below questions if you answered "No" to Question #1</u>.

6. Were you exposed to smoke from other people's cigarettes or tobacco products at home during childhood?
- ☐ Yes
  - If yes, hours per day? _____
- ☐ No

- ☐ Don't know
- ☐ Prefer not to answer

7. Were you exposed to smoke from other people's cigarettes or tobacco products at home during adulthood

- ☐ Yes
    - If yes, hours per day? _____
- ☐ No
- ☐ Don't know
- ☐ Prefer not to answer

8. Were you exposed to smoke from other people's cigarettes or tobacco products at work during adulthood?

- ☐ Yes
    - If yes, hours per day? _____
- ☐ No
- ☐ Don't know
- ☐ Prefer not to answer

Alcohol History:

1. Have you ever consumed alcoholic beverages, such as beer, wine, or liquor at least once a week for 6 months or more?
- ☐ Yes
- ☐ No
- ☐ Don't know
- ☐ Prefer not to answer

2. How often did you have a drink containing alcohol in the past 12 months? (Consider a "drink" to be a can or bottle of beer, a glass of wine, or 1 cocktail or shot of hard Liquor such as scotch, gin, or vodka.)
- ☐ None – Skip the remaining questions in this section
- ☐ Once a month or less
- ☐ 2 to 4 times a month
- ☐ 2 to 3 times a week
- ☐ 4 to 5 times a week
- ☐ 6 or more times a day

3. How many drinks did you have on a typical day when you were drinking in the past 12 months?
- ☐ 0 to 2 drinks
- ☐ 3 to 4 drinks
- ☐ 5 to 6 drinks
  - On more than one occasion?
    - ☐ Yes
    - ☐ No
- ☐ 7 to 9 drinks
  - On more than one occasion?
    - ☐ Yes
    - ☐ No
- ☐ 10 or more drinks
  - On more than on occasion
    - ☐ Yes
    - ☐ No

4. Did you ever regularly (more than once a week for a year) drink 3 or more alcoholic beverages in a day?

- ☐ Yes
  - If yes, what age did you start drinking regularly? ______
  - If yes and you stopped, what age did you stop drinking regularly? ______
- ☐ No

5. What do you drink mainly?

- ☐ Beer
- ☐ Wine
- ☐ Hard liquor

<u>Menstrual History</u>:

1. How old were you for your first menstrual period? _________

2. How old were you when you entered menopause? _______

3. Cause of Menopause:
- ☐ Natural
- ☐ Chemotherapy/medication induced
- ☐ Surgery on reproductive organs
- ☐ Other: ______________________

4. Between the ages of 18 and when you began menopause, have you ever experienced a time interval of 3 or more months when you did not have a menstrual period?
- ☐ Yes, one time only
- ☐ Yes, more than once
- ☐ No (skip to Question #7)
- ☐ Don't know (skip to Question #7)

5. If yes to Question #4, were you breastfeeding at the time?
- ☐ Yes
- ☐ No
- ☐ Don't know

6. If yes to Question #5, were you breastfeeding or pregnant every time this happened
- ☐ Yes
- ☐ No
- ☐ Don't know

7. During ages 18-22 what was the pattern of your menstrual cycles? (excluding time around pregnancies)

- ☐ Regular
- ☐ Usually irregular
- ☐ Always irregular
- ☐ No periods

8. Have you undergone mastectomy?

- ☐ Yes, one breast at age ____, Reason ______
- ☐ Yes, both breasts at age ____, Reason ______
- ☐ No

9. Have you undergone oophorectomy (removal of one or both ovaries)?

- ☐ Yes, Unilateral at age ____, Reason ______
- ☐ Yes, Bilateral at age ____ , Reason ______
- ☐ No

10. Have you undergone a hysterectomy (removal of the uterus)?

- ☐ Yes, at age ____, Reason_______
- ☐ No

11. Have you ever been pregnant?

- ☐ Yes
- ☐ No (skip to Question #16)

12. How many pregnancies have you had? __________

13. What was the outcome of your pregnancy?

	Pregnancy:														
**Outcome**	**1**	**2**	**3**	**4**	**5**	**6**	**7**	**8**	**9**	**10**	**11**	**12**	**13**	**14**	**15**
Live Birth															
Still Born															
Other															

☐ Other, please describe: ____

14. If live birth, did you breast feed?

☐ Yes

- If yes, how many months for each child?

____
____
____
____
____

☐ No

15. How old were you for your first birth? ___________

16. Have you ever taken hormone replacement therapy other than birth control pills (e.g. estrogen, estrogen/progesterone combination)?

☐ Yes

- If yes, how old were you when you first began taking any hormone therapy? _________

☐ No (skip the remaining questions in this section)

17. What type of therapy are you taking now or most recently took?

☐ Estrogen only
☐ Estrogen and progesterone (Provera or Prempro)
☐ Don't know

18. Are you still taking hormone therapy?

- ☐ Yes
- ☐ No
    - If no, how old were you when you stopped taking any hormone therapy? _____

19. Check all that apply, have you ever taken?

- ☐ Tamoxifen
- ☐ Raloxifene
- ☐ None
- ☐ Don't know

<u>Cancer History</u>:

1. Is your mother alive or deceased?
- ☐ Alive
  - What is her current age? ______
- ☐ Deceased
  - What was her age at death? _____

2. Did your mother ever have cancer?
- ☐ Yes
  - If yes, type of first cancer? __________
- ☐ No
- ☐ Don't know

3. Is your father alive or deceased?
- ☐ Alive
  - What is his current age? ______
- ☐ Deceased
  - What was his age at death? _____

4. Did your father ever have cancer?
- ☐ Yes
  - If yes, type of first cancer? __________
- ☐ No
- ☐ Don't know

5. How many brothers do you have? _______

6. How many sisters do you have? _______

7. Complete the following questions for **each sibling** (skip to the next question, if you do not have siblings):

- What is the current age, or if deceased their age at death?

  ____
  ____
  ____
  ____

- Did your (sister/brother) ever have cancer?

  ☐ Yes

  - If yes, type of first cancer (for each sibling)?

    ____
    ____
    ____
    ____

  - If yes, age at first cancer (for each sibling)?

    ____
    ____
    ____
    ____

  ☐ No
  ☐ Don’t know

8. How many daughters do you have? ______

9. How many sons do you have? _______

10. Complete the following questions for **each child** (skip to the next question, if you do not have children):

- What is the current age, or if deceased their age at death?
  ____
  ____
  ____
- Did your (daughter/son) ever have cancer?
  - o Yes
    - o If yes, type of first cancer (for each child)?
      ____
      ____
      ____
    - o If yes, age of first cancer (for each child)?
      ____
      ____
      ____
  - o No
  - o Don’t know

11. Have any of your close family members (parents, siblings, children) been diagnosed with or had a positive genetic test for the following hereditary cancer syndromes?

- ☐ None
- ☐ Lynch Syndrome (including MLH1, MSH2, MSH6 & PMS2 genes)
- ☐ Hereditary Breast/Ovarian Cancer syndrome (including BRCA1/2 genes)
- ☐ Cowden syndrome (PTEN gene)
- ☐ Familial adenomatous polyposis (APC gene)
- ☐ MUTYH-associated polyposis (MUTYH gene)
- ☐ Li-Fraumeni syndrome (TP53 gene)
- ☐ Hereditary melanoma (CDKN2A gene)
- ☐ Hereditary diffuse gastric cancer (CDH1 gene)
- ☐ Peutz-Jeghers syndrome (STK11 gene)
- ☐ Other hereditary cancer syndrome or positive genetic test for hereditary cancer (specify) _________

<u>Medications</u>:

Please check any medications that you ever used on a regular basis (i.e., at least once per week)

Check if YES		Less than 1 year	1 to 3 years	3 to 5 years	6 to 10 years	11 years or more
	Advil, Aleve, Motrin or other non-steroidal anti-inflammatory drugs					
	Celebrex, Vioxx of Bextra					
	Aspirin – full dose or extra strength					
	Aspirin – low dose or baby aspirin					
	Tylenol					
	Other drug for pain relief					

<u>Other Medical Conditions</u>: Please check any conditions that you have ever been diagnosed with.

**Have you ever been diagnosed with any of the following?**	**Yes**	**Year diagnosed?**	
Fibrocystic/ other benign breast disease			Confirmed by breast biopsy?<br>[ ] yes<br>[ ] no
			Confirmed by aspiration?<br>[ ] yes<br>[ ] no
Ductal Carcinoma in Situ			
Non-cancerous colon or rectal polyp (adenoma)			
Ulcerative colitis/Crohn's disease			
Gastric ulcer			
Barrett's esophagus			
Diabetes			Are you taking medicine for this?<br>[ ] yes<br>[ ] no<br>[ ] don't know
			If you are taking medicine, is it:<br>[ ] insulin<br>[ ] pills
			If you are taking medicine, at what age was this first treated?
			If you are taking insulin, was it your first diabetes medicine?<br>[ ] yes<br>[ ] no<br>[ ] don't know
			Did diabetes occur ONLY during pregnancy?<br>[ ] yes<br>[ ] no<br>[ ] don't know
Endometriosis- 1st diagnosis			Confirmed by laparoscopy?<br>[ ] yes<br>[ ] no
Uterine fibroids- 1st diagnosis			Confirmed by pelvic exam?<br>[ ] yes<br>[ ] no
			Confirmed by ultrasound/hysterectomy?<br>[ ] yes<br>[ ] no

**Have you ever been diagnosed with any of the following?**	**Yes**	**Year diagnosed?**	
Multiple sclerosis			
Emphysema/chronic bronchitis, doctor diagnosed			
Grave's disease/hyperthyroidism			
Thyroid nodule (benign)			
Gout			
SLE (systemic lupus)			
Rheumatoid arthritis, doctor diagnosed			
H. Pylori			
Hepatitis A			
Hepatitis B			
Hepatitis C			
Jaundice (yellowing of skin or eye)			
Cirrhosis			
Alcoholic liver disease			
Non-alcoholic fatty liver disease			
HIV (AIDS)			
Pancreatitis			Was this related to a procedure (ERCP or EUS)?<br><br>Did you have more than one episode?
Other major illness or surgery			Please specify:

1. During the past three years, have you had a chest x-ray?
   - ☐ Yes, once
   - ☐ Yes, more than once
   - ☐ No (skip to Question #3)
   - ☐ Don't know (skip to Question #3)

2. If yes, what was the outcome of the test?
   - ☐ Normal
   - ☐ Other, please explain: _______

3. During the past three years, have you had a test for blood in the stool?
   - ☐ Yes, once
   - ☐ Yes, more than once
   - ☐ No
   - ☐ Don't know

4. During the past three years, have you had a colonoscopy, sigmoidoscopy, virtual (CT) colonoscopy or barium enema to examine the colon and rectum?
   - ☐ Yes, once
   - ☐ Yes, more than once
   - ☐ Don't know
   - ☐ No

5. During the past three years, have you had an upper endoscopy to examine the esophagus, or stomach?
   - ☐ Yes, once
   - ☐ Yes, more than once
   - ☐ No
   - ☐ Don't know

6. During the past three years, have you had a mammogram?
   - ☐ Yes, once
   - ☐ Yes, more than once
   - ☐ No
   - ☐ Don't know

7. During the past three years, have you had a MRI of the breast?
   - ☐ Yes, once
   - ☐ Yes, more than once
   - ☐ No
   - ☐ Don't know

8. During the past three years, have you had a Pap smear?
   - ☐ Yes, once
   - ☐ Yes, more than once
   - ☐ No
   - ☐ Don't know

9. During the past three years, have you had a pelvic examination?
   - ☐ Yes, once
   - ☐ Yes, more than once
   - ☐ No
   - ☐ Don't know

10. During the past three years, have you had an ultrasound or scan of your ovaries?
    - ☐ Yes, once
    - ☐ Yes, more than once
    - ☐ No
    - ☐ Don't know

11. During the past three years, have you had a blood test for ovarian cancer (for example, CA-125)?
    - ☐ Yes, once
    - ☐ Yes, more than once
    - ☐ No
    - ☐ Don't know

12. During the past three years, have you had your pancreas screened?
    - ☐ Yes, via Computed Tomography (CT, CAT)
    - ☐ Yes, via Magnetic Resonance Imaging (MRI)
    - ☐ Yes, via Endoscopic Ultrasound (EUS)
    - ☐ Yes, via other screening method
    - ☐ No (skip to Question #14)

13. What was the outcome of the test?
    - ☐ Normal
    - ☐ Other, please explain: ________________

14. During the past three years, have you a Computed Tomography (CT, CAT)?
    - ☐ Yes
    - ☐ No (skip to Question #17)

15. Please indicate the reason for the CT/CAT scan: ________

16. Please indicate the results of the CT/CAT scan: ____________

17. During the past three years, have you an MRI?
    - ☐ Yes
    - ☐ No (skip the remaining questions in this section)

18. Please indicate the reason for the MRI: ________

19. Please indicate the results of the MRI: ____________
